# Molecular Diagnostics of Pathogenic *Vibrio*: Target Selection, Detection Platforms, and Challenges in Field Applications

**DOI:** 10.3390/ijms27198514

**Published:** 2026-09-24

**Authors:** Feng Liu, Yu Liu, Shuangle Jin, Qingbao Du

**Affiliations:** 1 Laboratory of Marine Ecology and Environmental Sciences, Institute of Oceanology, Chinese Academy of Sciences (IOCAS), Qingdao 266071, China; 2 Laboratory for Marine Ecology and Environmental Science, Qingdao Marine Science and Technology Center, Qingdao 266200, China; 3 College of Marine Science, University of Chinese Academy of Sciences, Beijing 101408, China; 4 Tsingtao SINOVA-HK Biotechnology Co., Ltd., Qingdao 266400, China

**Keywords:** pathogenic *Vibrio*, molecular diagnostics, target gene selection, nucleic acid amplification, CRISPR-Cas detection, isothermal amplification, multiplex detection, biosensors, point-of-care testing, foodborne pathogens

## Abstract

Pathogenic *Vibrio* species, including *Vibrio parahaemolyticus*, *Vibrio cholerae*, and *Vibrio vulnificus*, are major foodborne and environmental pathogens that threaten public health. Rapid and accurate detection is crucial for clinical diagnostics, food safety monitoring, and environmental surveillance. This review provides a comprehensive overview of molecular detection strategies for pathogenic *Vibrio*, integrating three key aspects: (i) selection of molecular targets, including species-specific, virulence-associated, and regulatory genes; (ii) analytical performance and limitations of major detection platforms, such as polymerase chain reaction (PCR)-based methods, isothermal amplification, clustered regularly interspaced short palindromic repeats (CRISPR)-CRISPR-associated (Cas) systems, high-throughput sequencing (HTS), and biosensor-based approaches; and (iii) applicability across clinical, food, and environmental scenarios. We highlight the influence of genetic variability, sample matrix complexity, and viability on detection accuracy and discuss the advantages and limitations of single- and multi-target approaches. Emerging trends toward next-generation diagnostics, including integrated microfluidic platforms, CRISPR-Cas-based detection, multiplex assays, and artificial intelligence(AI)-assisted data analysis, are also examined. By identifying gaps and challenges in the routine deployment of these technologies, this review offers practical insights for the development of rapid, field-deployable diagnostic strategies for *Vibrio* pathogens.

## 1. Introduction

Pathogenic *Vibrio* species, such as *Vibrio parahaemolyticus*, *Vibrio cholerae*, *Vibrio vulnificus*, *Vibrio alginolyticus*, and *Vibrio mimicus*, are significant foodborne and water-associated pathogens in marine and estuarine environments [1]. Human infections occur through the consumption of contaminated seafood, ingestion of polluted water, or exposure to seawater through open wounds [2,3,4]. These infections range from self-limiting gastroenteritis to severe conditions such as wound infections, septicemia, and death, particularly in immunocompromised individuals or those with underlying health conditions [5]. In recent years, factors such as climate change, warming coastal waters, ecological shifts in marine environments, and increased global seafood consumption have contributed to the wider distribution and rising incidence of *Vibrio* infections, challenging food safety, environmental monitoring, and public health surveillance [6,7].

Therefore, rapid and accurate detection is crucial for effective prevention, control, and risk assessment of pathogenic *Vibrio*. While conventional culture-based methods and biochemical identification techniques are still widely used due to their specificity, they are often limited by long turnaround times, labor-intensive procedures, and insufficient sensitivity in detecting low bacterial loads or stressed cells [8]. These limitations reduce their usefulness in early clinical diagnosis, timely food safety screening, and rapid response during outbreak investigations or environmental surveillance [9].

Molecular diagnostic approaches based on nucleic acid detection have become increasingly essential and are now central to detecting pathogenic *Vibrio*. Major detection platforms include conventional polymerase chain reaction (PCR), quantitative real-time PCR (qPCR), digital PCR (dPCR), and isothermal amplification methods like loop-mediated isothermal amplification (LAMP), recombinase polymerase amplification (RPA), and recombinase-aided amplification (RAA). RPA and RAA are collectively referred to as RPA/RAA, as well as emerging CRISPR-Cas-based systems. These molecular techniques generally offer enhanced analytical sensitivity, specificity, speed, and adaptability compared to traditional methods [10,11].

Despite rapid technological progress, no single molecular platform can fully meet the diverse requirements of all application scenarios. Techniques with high analytical sensitivity and strong quantitative capabilities, such as qPCR and dPCR, typically require specialized instruments and controlled laboratory environments. On the other hand, rapid and portable methods designed for field use, including LAMP, RPA/RAA, and lateral flow-based platforms, often face challenges like non-specific amplification, limited quantification, and reduced robustness in complex samples. Moreover, the diagnostic performance of a detection system is not solely determined by amplification efficiency or signal output. Factors like molecular target selection, sample matrix complexity, inhibitor interference, and contamination control significantly influence detection performance, especially in diverse contexts such as clinical diagnosis, food testing, environmental surveillance, and point-of-care testing (POCT). Balancing sensitivity, specificity, speed, portability, and practical applicability remains a central challenge [12].

A critical aspect of molecular detection is not only the analytical platform itself but also the biological relevance of the chosen molecular target. Species-specific genes, virulence-associated genes, and regulatory or functional markers vary significantly in diagnostic significance, stability, and discriminatory power. Single-target strategies may suffice for preliminary identification but are often inadequate for distinguishing pathogenic from non-pathogenic strains or addressing strain-level diversity in real-world samples. The increasing need for multiplex detection, on-site testing, and integrated analysis further complicates assay design and result interpretation [13,14].

Recent advances in the field are shifting focus from optimizing individual technologies to integrating complementary platforms. Examples include coupling isothermal amplification with CRISPR-Cas systems to enhance specificity, integrating nucleic acid amplification with lateral flow strips or biosensors for visual, portable readouts, and incorporating microfluidic devices with intelligent signal processing for automated detection [15]. These developments hold promise for achieving rapid, sensitive, and field-deployable detection systems for pathogenic *Vibrio*. However, significant bottlenecks remain, such as limited comparability across studies, insufficient standardization, challenges in multiplex assay design, and the gap between proof-of-concept studies and routine applications.

Existing reviews have largely focused on individual detection methods or specific platforms. Few have integrated molecular target selection, platform characteristics, and real-world application constraints into a unified analytical framework. To address this gap, this review provides a comprehensive and critical overview of molecular detection strategies for pathogenic *Vibrio*. We focus on three interconnected aspects: (i) the selection and diagnostic significance of molecular targets; (ii) the principles, advantages, and limitations of key detection platforms, including PCR-based methods, isothermal amplification, CRISPR-Cas-based systems, HTS, and biosensing technologies; and (iii) the applicability of these platforms in clinical, food, and environmental contexts. We also discuss current bottlenecks and emerging trends, including integrated detection systems, microfluidic platforms, intelligent data analysis, and multiplex pathogen detection. By linking molecular targets to detection performance and translational challenges, this review aims to provide a useful framework for developing rapid, accurate, and practical diagnostic strategies for pathogenic *Vibrio*.

This review maps the diagnostic value of molecular targets for the major pathogenic *Vibrio* species and compares detection platforms across clinical, food, and environmental matrices, with a special focus on the translational barriers to routine deployment. Our goal is to provide a comprehensive understanding of the practical challenges and emerging trends in *Vibrio* diagnostics, highlighting the limitations of existing technologies. An overview of pathogenic *Vibrio* diagnostics, including the key species, exposure contexts, analytical pillars, and future integration strategies, is presented in Figure 1. We also offer a roadmap for developing rapid, accurate, and field-deployable diagnostic tools, aimed at overcoming these challenges and improving the effectiveness of public health interventions.

## 2. Molecular Basis of *Vibrio* Detection

Molecular detection of pathogenic *Vibrio* relies fundamentally on the identification and amplification of specific nucleic acid targets. The selection of appropriate molecular markers is a critical determinant of diagnostic performance, directly influencing specificity, sensitivity, and the biological relevance of detection results. Unlike purely analytical systems, molecular diagnostics must consider not only assay efficiency but also the genetic diversity, evolutionary dynamics, and pathogenic mechanisms of the target organism. Therefore, understanding the molecular basis of target selection is essential for the rational design and interpretation of *Vibrio* detection strategies.

### 2.1. Classification of Molecular Targets

Molecular targets for *Vibrio* detection can be classified into four main categories: conserved genes, species-specific genes, virulence-associated genes, and regulatory or functional genes. Each category serves different diagnostic purposes and offers varying degrees of specificity and biological relevance [16,17].

Conserved genes, such as 16S rRNA, are useful for broad-spectrum bacterial detection and phylogenetic analysis, especially in metagenomic studies and environmental monitoring. However, their low discriminatory power at the species or strain level makes them inadequate for identifying closely related *Vibrio* species or assessing pathogenic potential [18].

Species-specific genes like *tlh* (*V. parahaemolyticus*), *ompW* (*V. cholerae*), and *vvhA* (*V. vulnificus*) offer higher specificity and are widely used in routine detection [19]. However, these markers cannot reliably differentiate between pathogenic and non-pathogenic strains, as many environmental isolates may carry these genes without expressing virulence [20,21].

Virulence-associated genes (e.g., *tdh*, *trh* for *V. parahaemolyticus*; *ctxA*, *ctxB* for *V. cholerae*) are crucial for assessing pathogenicity and are particularly relevant in food safety and epidemiological studies. However, their distribution can vary among strains, and their presence does not always correlate with actual pathogenicity, especially under varying environmental conditions [22,23].

Regulatory and functional genes, such as *toxR*, *rpoB*, and *gyrB*, provide additional diagnostic value when used in combination with other markers, aiding in strain-level identification and evolutionary studies [17,18]. However, these markers alone cannot indicate pathogenicity and are often more suited for phylogenetic or typing analysis.

### 2.2. Limitations of Single-Target Detection Strategies

Although single-target assays are widely used due to their simplicity and efficiency, they present inherent limitations in specificity, robustness, and interpretability, particularly in complex or field samples [24]. For instance, relying on a species-specific gene may result in false positives, especially in food safety, if non-pathogenic strains are present [25]. Similarly, relying solely on virulence genes for diagnosis may lead to false negatives if the target gene is absent or mutated in certain pathogenic strains.

Additionally, molecular detection systems based on nucleic acid amplification detect genetic material, not necessarily viable or infectious organisms [26]. In complex samples, such as food and environmental water, DNA from dead cells or extracellular DNA may persist, leading to overestimation of contamination risk. *Vibrio* species also exhibit viable but non-culturable (VBNC) states, further complicating the interpretation of molecular detection results [27].

Therefore, a more comprehensive approach using multi-target detection strategies is crucial for improving the reliability and specificity of *Vibrio* diagnostics. Multi-target assays can reduce false positives and negatives, enhance specificity, and provide complementary information when one target is compromised. Common nucleic acid targets for pathogenic *Vibrio* and their practical diagnostic relevance are summarized in Table 1.

### 2.3. Genetic Variability and Horizontal Gene Transfer

The genomic plasticity of *Vibrio* species presents a significant challenge for molecular detection. *Vibrio* genomes are highly dynamic, characterized by frequent genetic recombination, mutations, and horizontal gene transfer (HGT). Mobile genetic elements, such as plasmids, bacteriophages, and pathogenicity islands, play crucial roles in the dissemination of virulence genes and other functional traits [28].

This genetic variability can lead to false-negative or false-positive detection, as virulence genes may be gained or lost through horizontal gene transfer [29]. For instance, new strains that do not conform to traditional diagnostic criteria may emerge, complicating detection efforts. Additionally, sequence variations within target genes may affect primer binding and amplification efficiency, especially when assays do not account for genetic diversity across geographically distinct strains [30,31].

To address these challenges, strategies such as multi-target detection and using conserved and variable regions in combination can improve the robustness of molecular assays. Constant updates to primer/probe designs based on the latest genomic data are essential for maintaining assay reliability and ensuring that newly emerging strains are detected effectively.

### 2.4. Rationale for Multi-Target Detection

Given the limitations associated with individual molecular markers, multi-target detection strategies have become increasingly important in *Vibrio* diagnostics. By simultaneously targeting combinations of conserved, species-specific, and virulence-associated genes, multi-target assays can improve both specificity and reliability. For example, combining species identification markers with virulence genes allows differentiation between non-pathogenic and potentially pathogenic strains, while reducing the risk of misclassification [32].

Multi-target approaches also enhance robustness against genetic variation and technical failure. If one target is affected by mutation or inhibition, other targets can provide complementary information, reducing the likelihood of false results. This is particularly important in complex sample matrices, where amplification efficiency may vary across targets [33].

However, multi-target detection introduces additional challenges, including primer design complexity, potential competition among targets in multiplex reactions, and increased risk of cross-reactivity or non-specific amplification. Therefore, careful optimization and validation are required to ensure assay performance. The main diagnostic target classes and the rationale for multi-target testing are summarized in Figure 2.

In summary, the molecular basis of *Vibrio* detection extends beyond the choice of analytical platform and is fundamentally rooted in the selection and combination of appropriate genetic targets. A rational, context-dependent target selection strategy, combined with an understanding of genomic variability and diagnostic limitations, is essential for the development of reliable and practical detection systems.

## 3. Detection Platforms for Pathogenic *Vibrio*

Molecular detection of pathogenic *Vibrio* has evolved into a diverse set of analytical platforms, each with distinct principles, performance characteristics, and application constraints. While these platforms differ in amplification mechanisms, signal generation, and operational requirements, their diagnostic performance is ultimately shaped by the interplay between molecular target selection, assay design, and sample context. In this section, major detection platforms are discussed with emphasis on their underlying principles, analytical strengths, and practical limitations.

### 3.1. PCR-Based Methods

PCR-based methods, including conventional PCR, qPCR, and dPCR, remain the most established molecular detection techniques for pathogenic *Vibrio* [9,11,34]. While these methods offer high analytical sensitivity, specificity, and reproducibility, the reliability of qPCR in complex samples is frequently compromised by PCR inhibitors and inconsistent DNA extraction efficiencies. Such matrix-dependent limitations have been well-documented during the quantification of *V. alginolyticus*, *Listonella anguillarum*, and *Vibrio harveyi* in saline and microalgal environments, as well as in the detection of *V. parahaemolyticus* within naturally contaminated mussels [35,36]. Additionally, these methods require precise thermal cycling and specialized instrumentation, limiting their utility in field applications [37]. Although dPCR offers improved tolerance to inhibitors and higher sensitivity, its cost, low throughput, and technical complexity restrict its widespread use [38]. Advances in sample preparation methods and portable thermal cyclers are needed to make PCR more applicable for real-time, on-site detection of *Vibrio* [39].

### 3.2. Isothermal Amplification Technologies

Isothermal amplification technologies such as LAMP and RPA/RAA are highly attractive for field-deployable applications due to their ability to amplify nucleic acids at a constant temperature, eliminating the need for a specialized thermal cycler, and have been applied to *V. parahaemolyticus* using a fluorescence and colorimetric dual-mode LAMP assay [40,41]. LAMP provides high amplification efficiency and tolerance to inhibitors, while RPA/RAA enables rapid amplification at lower temperatures, making it suitable for portable devices and point-of-care testing. Specifically, RPA and reverse transcription RPA(RT-RPA) with lateral-flow readouts have enabled rapid detection of *V. parahaemolyticus*, while visual LAMP has been used to detect *V. vulnificus* in aquatic products and environmental water [14,42,43]. Despite these advantages, both methods face challenges, including the risk of non-specific amplification, primer design complexity, and limited quantitative capabilities [15,44]. These issues are particularly evident in complex sample matrices, where background DNA and inhibitors may compromise specificity and reproducibility. Developing more robust systems, such as integrated isothermal amplification platforms with CRISPR-Cas-based detection, could help address these limitations [14].

### 3.3. CRISPR-Cas-Based Detection Systems

CRISPR-Cas-based detection systems, including CRISPR-Cas12 and CRISPR-Cas13, offer powerful molecular diagnostic tools due to their high sequence specificity and ability to amplify detection signals, *Vibrio* specific examples include RPA–Cas13a for visual detection of *V. alginolyticus* and RPA–Cas12a for visual detection of *V. parahaemolyticus* [45,46,47]. These systems detect target sequences followed by collateral cleavage of reporter molecules, generating detectable signals in fluorescence, lateral flow, or electrochemical formats [48,49]. When combined with upstream amplification techniques like RPA or LAMP, CRISPR systems can enable rapid and highly specific detection, making them ideal for point-of-care applications, as illustrated by a 3D-printed handheld RPA–CRISPR-Cas12a device for *V. parahaemolyticus* [50,51]. However, challenges in standardization, reagent stability, and cost remain significant barriers to their large-scale implementation. Moreover, errors in the amplification stage can lead to non-specific signals, which may affect the overall diagnostic reliability [52]. Therefore, further optimization and standardization of both amplification and detection steps remain necessary. This need is reflected in recent *Vibrio* specific assays, including CE–RAA–CRISPR detection of *V. parahaemolyticus* in seafood, one-pot LAMP–Cas12a visual detection, and electrochemical Cas12a biosensing [53,54,55].

### 3.4. HTS and Chip-Based Technologies

HTS and gene chip-based technologies provide a comprehensive approach for the detection of pathogenic *Vibrio* by allowing simultaneous analysis of multiple targets, including species identification, virulence factors, and antimicrobial resistance genes. Microarray-based platforms have assessed genetic heterogeneity, antimicrobial resistance, and VBNC in human-pathogenic *Vibrio*, while metagenomic approaches have detected *V. cholerae* and *V. parahaemolyticus* sequence types and associated resistance and virulence genes in environmental and seafood samples [56,57,58]. HTS, such as next-generation sequencing (NGS), enables in-depth microbial community analysis, making it valuable for epidemiological studies and environmental monitoring, it has profiled low-abundance *Vibrio* and virulence factors in water and oysters, and mNGS has detected non-O1/non-O139 *V. cholerae* in a patient with hepatic space-occupying lesions [59,60]. However, these methods are typically limited by their high cost, complex workflows, and the need for extensive data analysis, making them less suitable for routine detection [61]. Gene chips, while capable of multiplex detection, also face challenges related to standardization, cost, and the need for specialized equipment. Despite these limitations, these technologies could nevertheless support large-scale *Vibrio* surveillance, source tracing, and outbreak investigations with efficient data processing tools and more affordable sequencing platforms.

### 3.5. Biosensors and Point-of-Care Detection Platforms

Biosensor-based detection platforms integrate biological recognition elements with physicochemical signal transduction, enabling rapid and portable detection of pathogenic *Vibrio*. These platforms, including electrochemical and optical sensors, offer the advantage of rapid detection, miniaturization, and compatibility with point-of-care settings [62,63,64,65,66]. When integrated with nucleic acid amplification or CRISPR-Cas-based detection, biosensors can provide high sensitivity and specificity [67,68]. However, the performance of biosensors can be affected by environmental conditions, sample composition, and device stability, leading to variability in detection results [69]. Further development is needed to improve sensor reliability and robustness, particularly in complex sample matrices. The integration of microfluidics and artificial intelligence for automated data analysis could enhance biosensor performance and facilitate real-time monitoring in field applications. The major molecular detection platforms for pathogenic *Vibrio* and their performance trade-offs are compared in Table 2.

### 3.6. Platform Integration and Emerging Trends

The integration of complementary technologies has become a focal point in the development of *Vibrio* detection systems [70,71]. Hybrid platforms that combine isothermal amplification with CRISPR-Cas-based detection or integrate biosensors with microfluidic devices offer high sensitivity, specificity, and portability in a single workflow [72]. Microfluidic platforms, in particular, have the potential to integrate sample preparation, nucleic acid amplification, and signal detection into compact systems, reducing reagent consumption and analysis time [73,74,75,76]. The integration of artificial intelligence for automated data analysis could further enhance the efficiency and user-friendliness of these systems [77]. However, challenges related to system complexity, reproducibility, and cost must be addressed before these integrated platforms can be widely adopted for routine field applications. The major molecular detection platforms for pathogenic *Vibrio* and their trade-offs are compared in Figure 3.

## 4. Cross-Platform Challenges in the Detection of Pathogenic *Vibrio*

Although a wide range of molecular detection platforms has been developed for pathogenic *Vibrio*, their performance is often evaluated under controlled laboratory conditions and may not directly translate to real-world applications. Across different platforms, several common challenges limit the reliability, comparability, and practical deployment of these technologies. These challenges arise not only from technical constraints, but also from the intrinsic complexity of biological samples and the dynamic nature of *Vibrio* populations.

### 4.1. Sample Matrix Complexity and Inhibitor Interference

One of the most critical factors affecting the detection performance of *Vibrio* species is the complexity of sample matrices. Pathogenic *Vibrio* are typically detected in diverse sample types, such as seafood, environmental water, and clinical specimens, which contain substances that can interfere with nucleic acid extraction and amplification [78]. Common inhibitors, such as polysaccharides, proteins, and salts, can significantly reduce amplification efficiency, leading to false negatives or compromised results. While platforms like dPCR and certain LAMP assays demonstrate improved tolerance to inhibitors, no method is fully immune to matrix effects. The efficiency of nucleic acid extraction and purification also varies widely across protocols, which further impacts detection consistency. To address these challenges, the development of robust, extraction-free or simplified workflows, combined with efficient inhibitor removal strategies, is essential for improving reproducibility and enabling field-deployable solutions [79].

### 4.2. Sensitivity, Specificity, and Practical Trade-Offs

A fundamental challenge in *Vibrio* detection is the trade-off between analytical performance (sensitivity and specificity) and practical applicability. Highly sensitive methods such as qPCR and dPCR are ideal for detecting low-abundance targets but require specialized equipment, controlled laboratory environments, and skilled personnel, which limits their field applicability. In contrast, rapid and portable methods like LAMP, RPA/RAA, and lateral flow assays offer operational simplicity and fast turnaround times, but often sacrifice sensitivity and quantitative capability. These trade-offs are further compounded by the specific needs of each application scenario. For example, clinical diagnostics may prioritize sensitivity, while environmental monitoring or food safety screening may demand rapid, high-throughput, and cost-effective methods. Therefore, selecting the most suitable platform requires careful consideration of the trade-offs and the context in which it will be used.

### 4.3. Viability and Biological Relevance of Detection Signals

Molecular detection methods primarily detect genetic material rather than viable or infectious organisms. This distinction becomes particularly problematic in the context of *Vibrio* detection, where DNA from dead cells or extracellular sources may persist in samples, leading to potential overestimation of contamination risk. Additionally, *Vibrio* species can enter a VBNC state under environmental stress, where cells remain metabolically active but are not detected by conventional culture methods [80,81]. As a result, molecular detection may provide positive signals that do not necessarily correlate with the risk of infection. To improve the biological relevance of detection signals, approaches such as viability PCR, RNA-based detection, and culture-based confirmation are emerging, though no standardized solution has been widely adopted [82]. Further development of these methods is needed to bridge the gap between molecular signals and actual infection potential, particularly for food safety and environmental monitoring.

### 4.4. Limitations of Multiplex Detection

The increasing demand for simultaneous detection of multiple targets, such as various *Vibrio* species and virulence markers, has led to the development of multiplex assays. While multiplex PCR, multi-channel CRISPR-Cas systems, and high-throughput platforms allow for the detection of multiple targets simultaneously, these systems also introduce additional technical challenges. Primer and probe design complexity increases with the number of targets, raising the risk of cross-reactivity, primer-dimer formation, and amplification competition, which can compromise sensitivity and complicate quantitative analysis [83]. In isothermal amplification systems, multiplexing poses particular challenges due to the reliance on multiple primers, increasing the likelihood of non-specific reactions [84]. Despite these issues, multiplex detection is essential for food safety and environmental monitoring, as it allows for simultaneous detection of multiple pathogens and genes, improving diagnostic efficiency. Future research should focus on optimizing multiplex systems to minimize cross-reactivity and enhance assay reliability [85].

### 4.5. Lack of Standardization and Cross-Study Comparability

A major limitation in the field of *Vibrio* detection is the lack of standardized protocols and evaluation criteria across studies. Variations in sample preparation, target selection, amplification conditions, and detection thresholds often make it difficult to directly compare results from different studies. Additionally, many studies use artificially spiked samples or laboratory strains, which may not accurately reflect the complexity of real-world samples. The absence of standardized reference materials and validation frameworks further complicates the translation of research findings into practical applications. Establishing unified guidelines for assay design, performance evaluation, and reporting would significantly enhance comparability across studies and facilitate the development of reliable detection systems that can be widely implemented in clinical, environmental, and food safety settings.

### 4.6. Challenges in Technology Translation and Field Deployment

Despite significant advances in molecular detection technologies, the transition from laboratory-based research to routine field deployment remains a major challenge. Many high-performance methods are demonstrated under controlled conditions, but face significant barriers when applied in real-world settings. Key challenges include reagent stability under non-ideal storage conditions, high instrumentation and consumable costs, and the need for trained personnel to operate complex systems. Moreover, for field applications, such as point-of-care testing or on-site environmental monitoring, additional constraints such as portability, power supply, ease of use, and robustness are critical. While integrated platforms that combine amplification, detection, and readout are actively being developed, achieving reliable, cost-effective, and scalable “sample-to-result” systems remains a significant hurdle [86]. Addressing these challenges requires innovative approaches to system design, standardization, and cost reduction.

## 5. Future Perspectives

Future development of molecular detection strategies for pathogenic *Vibrio* is expected to move beyond incremental improvements in individual technologies toward the integration of molecular, analytical, and engineering approaches. Addressing the limitations identified in current platforms requires not only enhancing analytical performance, but also improving robustness, standardization, and real-world applicability.

### 5.1. Integration of Amplification and CRISPR-Cas-Based Recognition

One of the most promising directions for future *Vibrio* diagnostics is the integration of nucleic acid amplification with CRISPR-Cas-based recognition systems. While isothermal amplification methods such as LAMP and RPA/RAA allow rapid signal generation, CRISPR-Cas systems add an extra layer of sequence-specific recognition, significantly improving specificity and reducing false-positive results. Future research should focus on optimizing the coupling between amplification and CRISPR-Cas recognition to minimize errors from upstream amplification. Strategies like amplification-free CRISPR-Cas detection and improved guide RNA design can further enhance assay reliability. Additionally, simplifying workflows and reducing system complexity will be crucial for making these integrated technologies practical for routine and field applications.

### 5.2. Development of Integrated and Automated Detection Platforms

The future of *Vibrio* detection lies in the transition from multi-step laboratory procedures to integrated “sample-to-result” systems. Microfluidic technologies are a promising solution for integrating sample preparation, nucleic acid extraction, amplification, and detection into compact, automated platforms. These integrated systems should prioritize automation and minimal user intervention, making them ideal for rapid, decentralized field applications. However, challenges such as fluid control, reaction compatibility, and device standardization must be addressed to ensure reliable performance. By combining microfluidics with portable detection devices and cloud-based data processing, we can enable real-time, user-friendly diagnostics for *Vibrio* detection in various field settings.

### 5.3. Strategies for Improving Detection in Complex Samples

Improving detection in complex samples, such as seafood, environmental water, and clinical specimens, requires addressing the challenges posed by sample matrix complexity and inhibitor interference. Future approaches could include developing robust sample pretreatment methods, extraction-free detection systems, and engineered enzymes with enhanced tolerance to inhibitors. Advances in materials science, such as the use of nanomaterials for selective enrichment and signal enhancement, could also contribute to improved performance in complex matrices [87]. Incorporating internal controls and normalization strategies will further enhance the reliability of detection results, making them more suitable for real-world applications.

### 5.4. Advancing Multiplex and High-Throughput Detection

With the increasing need for simultaneous detection of multiple pathogens and virulence markers, multiplex detection will become increasingly important. Future developments should focus on enhancing multiplex capacity while maintaining sensitivity and specificity. Innovations in primer/probe design, spatial separation strategies, and multi-channel detection systems, including CRISPR-Cas-based multiplex platforms and microfluidic arrays, will drive advancements in multiplex diagnostics. However, the interpretation of complex multiplex data requires the development of advanced data analysis frameworks to reduce ambiguity and ensure accurate results. Improving the handling of multiple targets simultaneously is critical for broad applications in clinical diagnostics and environmental monitoring.

### 5.5. Enhancing Biological Relevance of Detection Signals

Future advances in *Vibrio* diagnostics should focus on improving the biological relevance and practical interpretability of molecular detection results. Rather than pursuing sensitivity alone, greater attention should be directed toward viability-informed detection workflows and the integration of molecular assays with complementary confirmation strategies, including culture-based or functional validation where appropriate [81]. Such developments would support more context-aware interpretation of molecular findings in food and environmental surveillance [88].

In addition, future work should place greater emphasis on method optimization in complex sample matrices, inter-laboratory standardization, and real-world performance evaluation under field-relevant conditions. Progress in these areas will be important for improving comparability across studies and facilitating broader routine application of these methods in surveillance and public health practice.

### 5.6. Standardization and Validation Frameworks

The lack of standardized protocols and evaluation criteria remains a major barrier to the routine use of *Vibrio* detection technologies. Efforts should focus on establishing unified standards for assay design, performance evaluation, and reporting. The development of standardized reference materials, inter-laboratory validation studies, and regulatory guidelines will enhance comparability across studies and ensure the reliability of detection systems. Collaboration between researchers, industry stakeholders, and regulatory bodies is essential for creating these frameworks and facilitating the translation of research into practical applications.

### 5.7. Integration of Artificial Intelligence and Data-Driven Analysis

As detection platforms become more complex and data outputs grow more detailed, artificial intelligence (AI) and machine learning will play a crucial role in future diagnostics [89]. AI can assist in signal interpretation, pattern recognition, and anomaly detection, especially in multiplex and high-throughput systems [90]. Data-driven models may also enable predictive analysis of contamination trends and support risk assessment [91,92]. Integrating AI with portable detection systems could provide real-time decision-making and automated interpretation, reducing reliance on expert analysis and increasing the accessibility of diagnostics [93]. However, challenges related to data quality, model validation, and interpretability must be addressed before these systems can be widely implemented.

### 5.8. Toward Practical and Scalable Detection Systems

The ultimate success of molecular detection technologies for pathogenic *Vibrio* will depend on their ability to meet the practical requirements of real-world applications. Future systems must balance sensitivity, specificity, cost, speed, and ease of use. Efforts should focus on simplifying workflows, reducing costs, and improving reagent stability to make these systems more suitable for diverse application scenarios. Scalable manufacturing, user-friendly design, and streamlined regulatory approval processes will be critical for translating laboratory innovations into widely adopted diagnostic tools that can be deployed globally. The major barriers to reliable, field-deployable *Vibrio* diagnostics and the corresponding future responses are shown in Figure 4.

## 6. Conclusions

The molecular detection of pathogenic *Vibrio* has evolved significantly, moving from conventional PCR-based assays to more advanced platforms, including isothermal amplification, CRISPR-Cas-based systems, HTS, and biosensor-integrated technologies. These innovations have greatly enhanced analytical sensitivity, specificity, and speed, enabling broader applications in clinical diagnostics, food safety monitoring, and environmental surveillance [1,34].

However, the effectiveness of these technologies extends beyond analytical performance alone. As discussed in this review, factors such as molecular target selection, genetic variability, sample matrix complexity, and the intended application context play pivotal roles in determining the reliability and practical utility of *Vibrio* detection. No single detection platform can simultaneously meet all the requirements for sensitivity, specificity, speed, cost, and portability. The field is thus characterized by inherent trade-offs, necessitating context-dependent selection and careful interpretation of results.

A key insight from this review is that the integration of complementary strategies—such as combining appropriate molecular targets with suitable detection platforms—is crucial for improving diagnostic accuracy and robustness. Multi-target detection strategies can enhance specificity and mitigate the effects of genetic variability, while integrated systems that combine amplification, CRISPR-Cas-based recognition, and portable readout technologies offer promising pathways for the development of rapid, field-deployable diagnostic solutions.

Despite the substantial progress made, several critical challenges remain. These include the lack of standardized protocols and evaluation frameworks, limited comparability across studies, challenges in interpreting molecular signals in terms of biological relevance, and obstacles in translating laboratory-based technologies to routine, field-deployable applications. Addressing these challenges will require coordinated efforts to enhance assay design, establish unified evaluation criteria, and bridge the gap between analytical innovations and practical deployment.

Looking forward, the future of pathogenic *Vibrio* detection will rely on the development of integrated, intelligent, and application-oriented systems. Advances in microfluidics, biosensing, multiplex detection, and artificial intelligence are expected to play central roles in this transition. However, the ultimate success of these technologies will depend on their ability to operate reliably under real-world conditions, provide biologically meaningful results, and meet the practical needs of various use scenarios.

In conclusion, a deeper integration of molecular understanding, technological innovation, and application-driven design is essential for advancing the field. This approach will foster the development of rapid, accurate, and scalable detection systems, ultimately enabling more effective control of pathogenic *Vibrio* in food safety, environmental monitoring, and public health management.

## Figures and Tables

**Figure 1:**
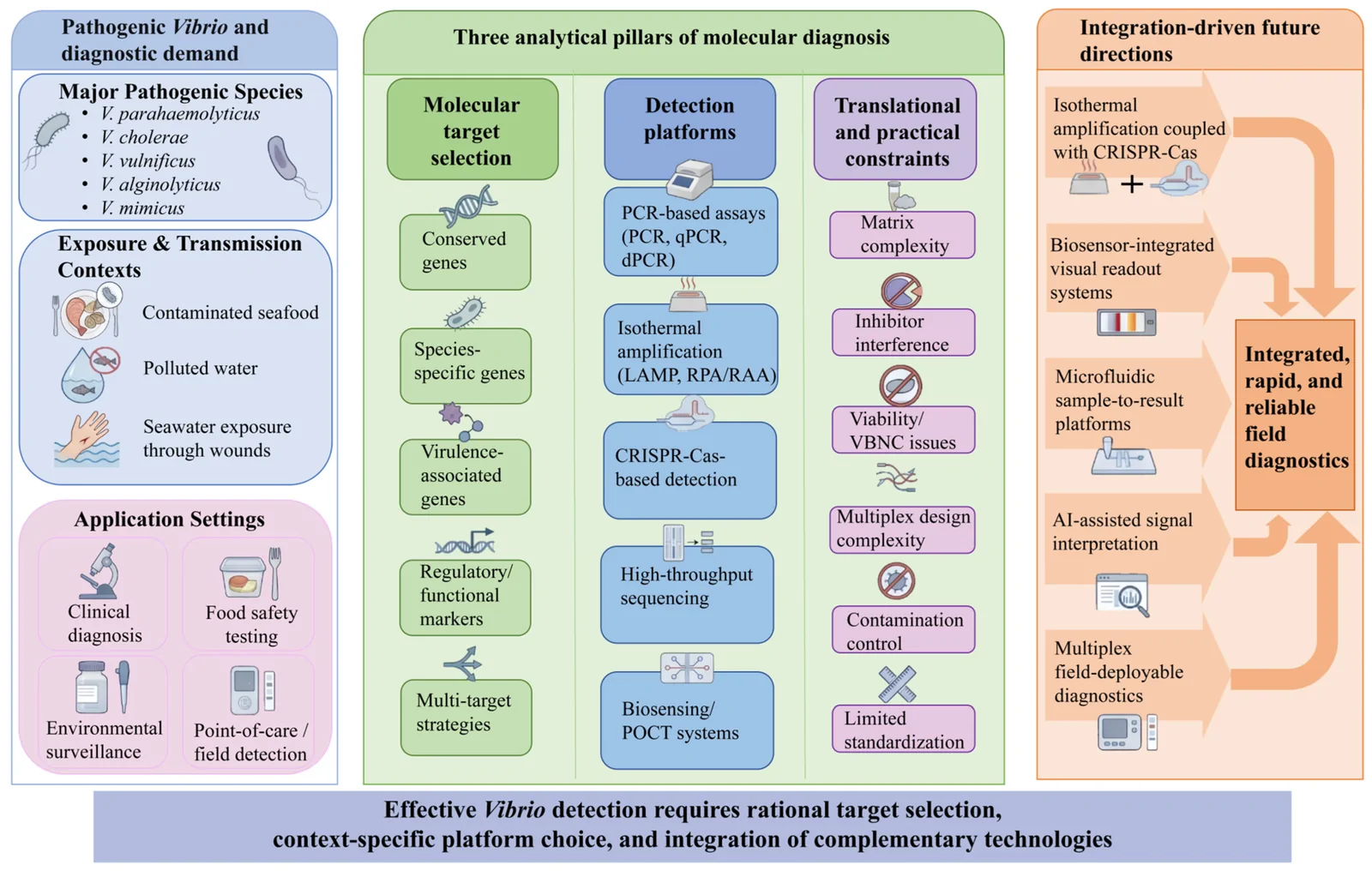
Overview of pathogenic *Vibrio* diagnostics, spanning major species, exposure contexts, analytical pillars, and integration-driven future directions. This figure summarizes the clinically relevant pathogenic *Vibrio* species, common exposure and transmission settings, the principal analytical pillars underpinning molecular diagnosis, and emerging integration strategies aimed at improving speed, robustness, and field deployability.

**Figure 2:**
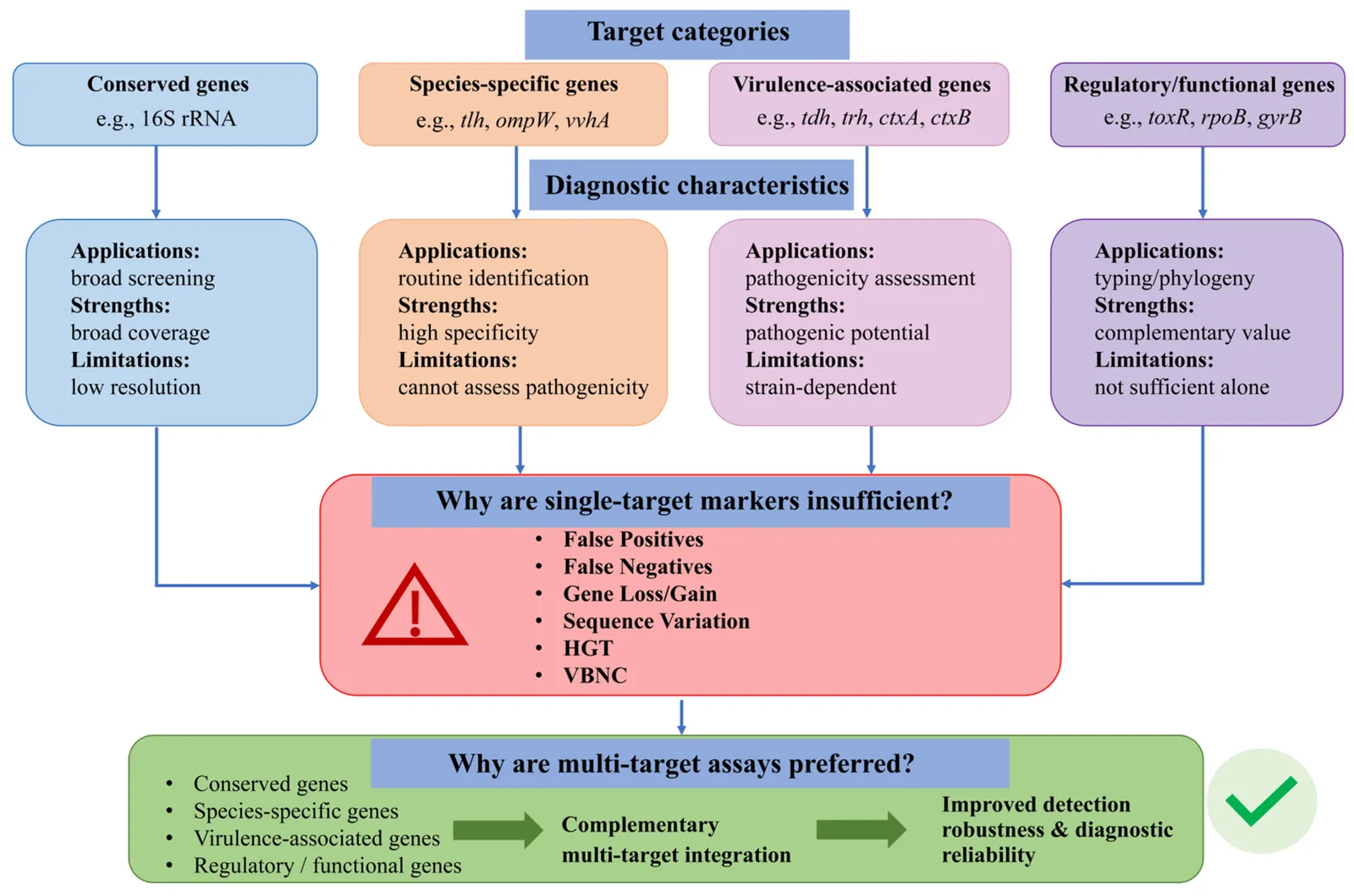
Rationale for multi-target molecular marker selection in pathogenic *Vibrio* detection. This figure shows the major categories of molecular markers used for pathogenic *Vibrio* detection, including conserved genes, species-specific genes, virulence-associated genes, and regulatory/functional markers. It emphasizes why multi-target panels are more robust than single-target assays against sequence variation, HGT, and VBNC-related limitations.

**Figure 3:**
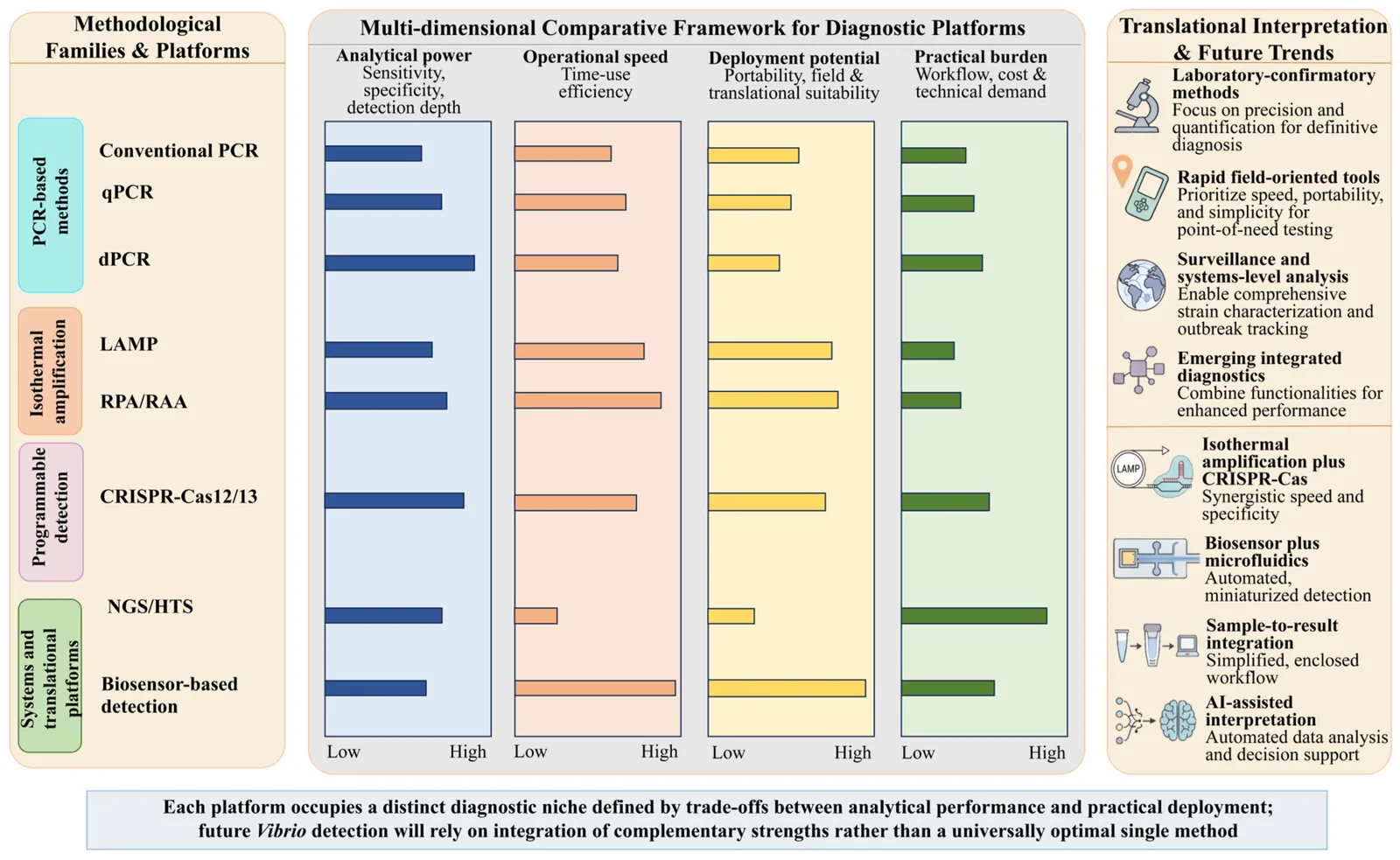
Comparative framework of major molecular detection platforms for pathogenic *Vibrio*. This figure compares PCR, qPCR, dPCR, LAMP, RPA/RAA, CRISPR-Cas-based assays, NGS/HTS, and biosensor-based systems in terms of analytical performance, speed, deployability, and practical burden. It also points to future development through amplification-CRISPR integration, biosensor–microfluidic coupling, sample-to-result workflows, and AI-assisted interpretation.

**Figure 4:**
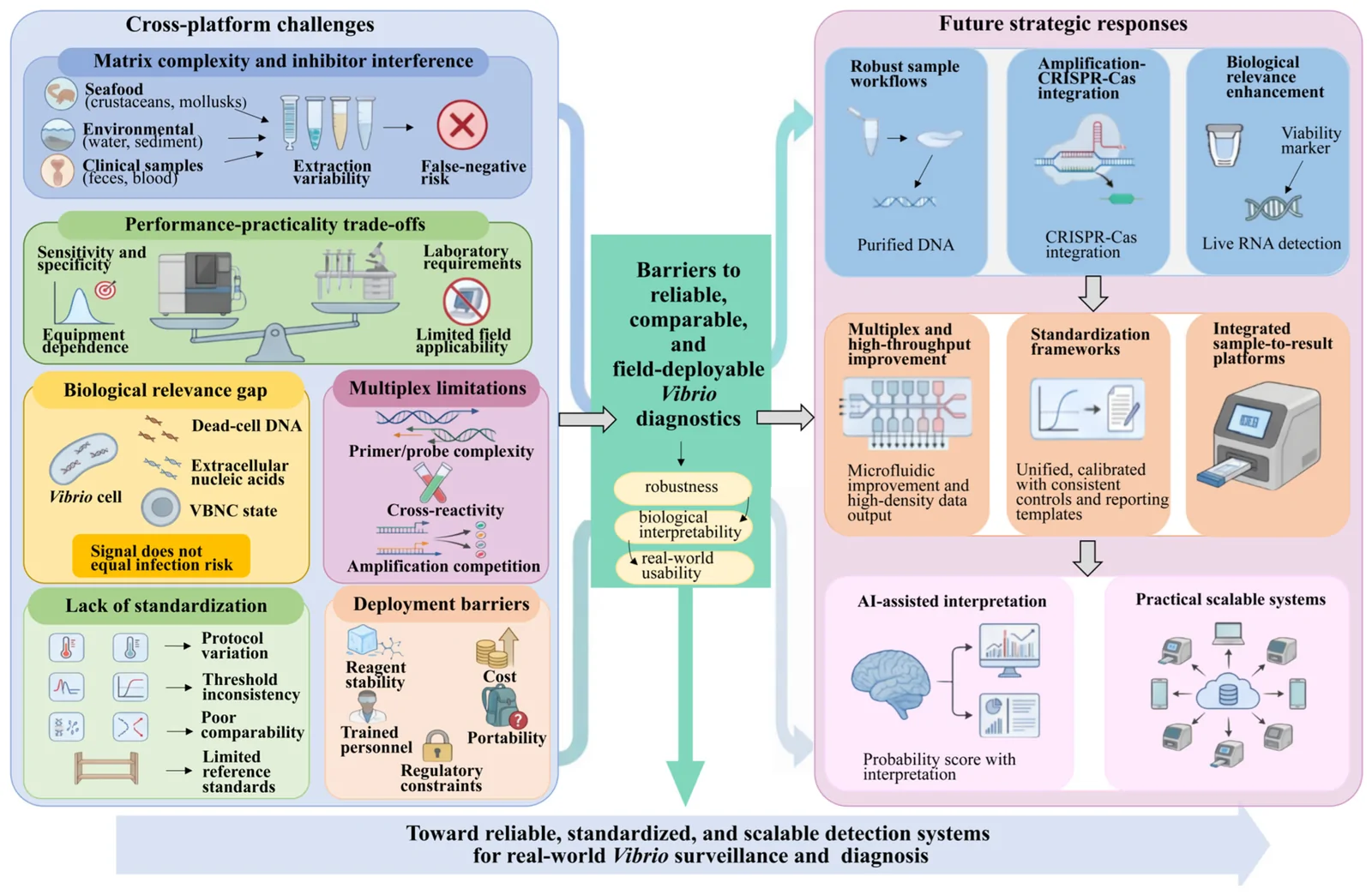
Cross-platform challenges and future strategic responses for real-world *Vibrio* molecular diagnostics. This figure summarizes the main barriers to real-world *Vibrio* detection, including matrix effects, inhibitor interference, dead-cell DNA, VBNC states, multiplexing complexity, limited standardization, and deployment constraints. It also outlines corresponding solutions such as robust sample workflows, viability-linked detection, multiplex optimization, standardized frameworks, integrated sample-to-result platforms, and AI-assisted interpretation.

**Table 1:** Common Nucleic Acid Targets for Pathogenic *Vibrio* and Their Practical Relevance.

*Vibrio* Species	Target Gene	Gene Type	Function/Role	Practical Relevance	Limitations/Notes
*V. parahaemolyticus*	*tlh*	Species-specific	Thermolabile hemolysin	Species identification; basic PCR screening	Single-target may miss atypical strains; cannot indicate virulence
*V. parahaemolyticus*	*tdh*	Virulence	Thermostable direct hemolysin	Pathogenicity assessment; food and clinical testing	Gene absent in some pathogenic isolates
*V. parahaemolyticus*	*trh*	Virulence	TDH-related hemolysin	Auxiliary virulence evaluation; risk assessment	Variable distribution among strains; supplementary marker
*V. parahaemolyticus*	*toxR*	Regulatory	Virulence regulatory protein	Strain typing; auxiliary detection	Cannot directly indicate pathogenicity; sequence variability exists
*V. parahaemolyticus*	*gyrB*	Functional	DNA gyrase	Phylogenetic analysis; molecular epidemiology	Conserved gene; not directly linked to virulence
*V. cholerae*	*ompW*	Species-specific	Outer membrane protein	Rapid species identification; routine PCR/qPCR	Cannot differentiate pathogenic vs. non-pathogenic strains
*V. cholerae*	*ctxA/ctxB*	Virulence	Cholera toxin subunits	Pathogenicity confirmation; clinical testing	May be absent in some toxigenic strains; horizontal transfer possible
*V. cholerae*	*tcpA*	Virulence	Toxin-coregulated pilus	Colonization potential assessment; pathogenic mechanism research	Not universally present in all strains
*V. cholerae*	*toxR*	Regulatory	Virulence regulatory system	Auxiliary detection; molecular surveillance	Sequence variability may affect assay performance
*V. vulnificus*	*vvhA*	Species-specific	Hemolysin	Pathogenicity marker; clinical testing	Single marker may miss atypical pathogenic strains
*V. vulnificus*	*vcg*	Typing	Clinical/environmental typing	Virulence typing; risk assessment	Limited use for direct detection; mainly epidemiological
Universal *Vibrio* markers	16S rRNA	Conserved	Ribosomal RNA; phylogenetic marker	Broad-spectrum detection; preliminary screening; metagenomics	Low species-level resolution; cannot indicate virulence
Universal *Vibrio* markers	*rpoB*	Functional	RNA polymerase	Taxonomic identification; typing analysis	Conserved across strains; limited virulence information
Universal *Vibrio* markers	*gyrB*	Functional	DNA gyrase	Evolutionary analysis; molecular epidemiology	Sequence conservation limits strain discrimination
Multi-target combinations	*tlh* + *tdh* + *trh*	Combined	Species + virulence	Improved diagnostic accuracy; food testing	Requires multiplex optimization; primer interactions possible
Multi-target combinations	*ompW* + *ctxA*	Combined	Species identification + virulence	High-specificity detection; clinical/environmental testing	Complexity increases assay setup; potential cross-reactivity
Multiplex PCR target combinations	Multiple genes	Multi-target	Simultaneous detection	High-throughput screening; multi-pathogen surveillance	Requires careful primer design; non-specific amplification risk

**Table 2:** Molecular Detection Platforms for Pathogenic *Vibrio*.

Platform	Target Genes	Sample Type	Sensitivity (LOD)	Detection Time	Multiplex Capability	Key Limitations	Representative References
Conventional PCR	Species-specific (e.g., *tlh*, *ompW*, *vvhA*)	Seafood, water	10^3^–10^4^ copies/reaction	3–6 h	Low	Requires thermal cycler, inhibitor-sensitive, low throughput	[9,13]
qPCR	Species-specific & virulence genes (e.g., *tdh*, *trh*, *ctxA*)	Clinical, food	10–100 copies/reaction	2–4 h	Medium	Expensive instrumentation, lab-dependent, inhibitor interference	[14,15]
dPCR	Species-specific & virulence genes	Food, clinical	1–10 copies/reaction	2–5 h	Medium	High cost, low throughput, complex workflow	[8,23]
LAMP	Species-specific/virulence genes	Seafood, water	10^2^–10^3^ copies/reaction	30–60 min	Medium	Non-specific amplification, primer design complexity	[45,48]
RPA/RAA	Species-specific/virulence genes	Food, environmental	10–100 copies/reaction	20–40 min	Medium	Low quantification ability, prone to contamination	[46,50]
CRISPR-Cas12/Cas13	Species-specific + virulence genes	Food, water, clinical	1–10 copies/reaction (with pre-amplification)	30–60 min	High	Upstream amplification required, reagent stability, integration complexity	[55,56]
NGS/HTS	Conserved, species-specific, virulence, regulatory	Complex microbial communities, environmental	1–10 copies/reaction	1–3 days	Very High	Expensive, data analysis intensive, cannot distinguish viable vs. non-viable cells	[57,59]
Biosensor-based detection	Species-specific/virulence genes	Seafood, environmental, point-of-care	10^2^–10^3^ copies/reaction	10–60 min	Medium	Signal variability, matrix interference, integration challenges	[60,62]

## Data Availability

The original contributions presented in this study are included in the article. Further inquiries can be directed to the corresponding author.

## Abbreviations

The following abbreviations are used in this manuscript:

16S rRNA	16S ribosomal RNA
AI	Artificial intelligence
Cas	CRISPR-associated protein
Cas12a	CRISPR-associated protein 12a
Cas13a	CRISPR-associated protein 13a
CRISPR	Clustered regularly interspaced short palindromic repeats
*ctxA*	Cholera toxin A subunit gene
*ctxB*	Cholera toxin B subunit gene
dPCR	Digital PCR
DNA	Deoxyribonucleic acid
*gyrB*	DNA-directed RNA polymerase subunit beta gene
HGT	Horizontal gene transfer
HTS	High-throughput sequencing
LAMP	Loop-mediated isothermal amplification
LOD	Limit of detection
mNGS	Metagenomic next-generation sequencing
NGS	Next-generation sequencing
*ompW*	Outer membrane protein W gene
PCR	Polymerase chain reaction
POCT	Point-of-care testing
qPCR	Quantitative real-time PCR
RAA	Recombinase-aided amplification
RNA	Ribonucleic acid
RPA	Recombinase polymerase amplification
RPA-CRISPR	Recombinase polymerase amplification–CRISPR assay
*rpoB*	DNA-directed RNA polymerase subunit beta
RT-RPA	Reverse transcription RPA
*tcpA*	Toxin-coregulated pilus A gene
*tdh*	Thermostable direct hemolysin gene
*tlh*	Thermolabile hemolysin gene
*toxR*	ToxR regulatory gene
*trh*	tdh-related hemolysin gene
VBNC	Viable but non-culturable
vcg	Virulence-correlated gene
*vvhA*	*Vibrio vulnificus* hemolysin A gene
*V. alginolyticus*	*Vibrio alginolyticus*
*V. cholerae*	*Vibrio cholerae*
*V. parahaemolyticus*	*Vibrio parahaemolyticus*
*V. vulnificus*	*Vibrio vulnificus*
